# Central Apneas Due to the CLIFAHDD Syndrome Successfully Treated with Pyridostigmine

**DOI:** 10.3390/ijerph19020775

**Published:** 2022-01-11

**Authors:** Anna Winczewska-Wiktor, Adam Sebastian Hirschfeld, Magdalena Badura-Stronka, Irena Wojsyk-Banaszak, Paulina Sobkowiak, Alicja Bartkowska-Śniatkowska, Valeriia Babak, Barbara Steinborn

**Affiliations:** 1 Chair and Department of Developmental Neurology, Poznan University of Medical Sciences, Przybyszewskiego 49, 60-355 Poznan, Poland; bstein@ump.edu.pl; 2 Chair and Department of Medical Genetics, Poznan University of Medical Sciences, 60-352 Poznan, Poland; adamhirschfeldmd@gmail.com (A.S.H.); badurastronka@ump.edu.pl (M.B.-S.); valeriana.babak@ump.edu.pl (V.B.); 3 Centers of Medical Genetics GENESIS, 60-529 Poznan, Poland; 4 Department of Pulmonology, Pediatric Allergy and Clinical Immunology, Poznan University of Medical Sciences, 60-572 Poznan, Poland; iwojsyk@ump.edu.pl (I.W.-B.); paulinasobkowiak@ump.edu.pl (P.S.); 5 Department of Pediatric Anesthesiology and Intensive Therapy, Poznan University of Medical Sciences, 60-572 Poznan, Poland; asniatko@ump.edu.pl

**Keywords:** CLIFAHDD, pyridostigmine, *NALCN*, apnea

## Abstract

*NALCN* mutations lead to complex neurodevelopmental syndromes, including infantile hypotonia with psychomotor retardation and characteristic facies (IHPRF) and congenital contractures of limbs and face, hypotonia, and developmental delay (CLIFAHDD), which are recessively and dominantly inherited, respectively. We present a patient in whom congenital myasthenic syndrome (CMS) was suspected due to the occurrence of hypotonia and apnea episodes requiring resuscitation. For this reason, treatment with pyridostigmine was introduced. After starting the treatment, a significant improvement was observed in reducing the apnea episodes and slight psychomotor progress. In the course of further diagnostics, CMS was excluded, and CLIFAHDD syndrome was confirmed. Thus, we try to explain a possible mechanism of clinical improvement after the introduction of treatment with pyridostigmine in a patient with a mutation in the *NALCN* gene.

## 1. Introduction

The excitability of neurons is significantly dependent on their ion channel complexes. In this group, the *NALCN* channels fulfill an essential part of the preservation of the resting membrane potential [1]. Notably, some clinical and genetic reports described pathogenic variants affecting the function of human *NALCN* protein and its Unc80 ancillary subunit as adverse causes of complex neurodevelopmental syndromes [2,3]. Recessive pathogenic variants were associated with infantile hypotonia with psychomotor retardation and characteristic facies (IHPRF) type 1 (OMIM #615419) and type 2 (OMIM #616801) syndromes. On the other hand, the dominant pathogenic variants were linked to the congenital contractures of limbs and face, hypotonia, and developmental delay syndrome (CLIFAHDD; OMIM #616266). Both IHPRF and CLIFAHDD patients present hypotonia, facial dysmorphisms, global developmental delay, or respiratory abnormalities. Nonetheless, in CLIFAHDD and IHPRF, the electrophysiological and functional consequences of the *NALCN* channel impairment remain uncertain. This report presents a patient with CLIFAHDD syndrome who was initially suspected of having congenital myasthenic syndrome (CMS). Due to the primary suspicion of CMS, the patient was started on pyridostigmine treatment. We describe the surprisingly positive clinical effect of such therapy and characterize its potential beneficial mechanism in patients with CLIFAHDD.

## 2. Case Description

The presented male patient was in neurological care in the first weeks of life due to the observed apnea episodes. Until the age of 3 months, the child’s development and the neurological examination results did not raise any concerns except for persistent episodes of apnea (which were treated as breath-holding spells) and symptoms of gastroesophageal reflux. The attempt of ferrum supplementation did not affect the number of episodes but intensified constipation. For this reason, the treatment was discontinued. Since the age of 3 months, the first symptoms of developmental delay were observed. There was no proper control of the head position and a significant decrease in muscle tension in the long body axis. Correct movement patterns were observed in the limbs, with proper muscle tension and reflexes.

Despite treatment for gastroesophageal reflux, symptoms worsened over time. After 7 months of age, apneas lasted for up to 60 s. At 12 months of age, incidents of respiratory insufficiency were prolonged to 3 min and required ventilatory support. Moreover, episodes of O2 saturation being decreased to 70%, without accompanying clinical symptoms, were observed at night. Spinal muscular atrophy was excluded by MLPA of the *SMN1* gene and familial dysautonomia by Sanger sequencing of the *ELP1* gene. The presence of distal arthrogryposis, hypotonia, and central apneas raised suspicion of congenital myasthenic syndrome (e.g., related to variants in *RAPSN* or *CHAT* gene). As symptoms rapidly aggravated, it was decided to initiate treatment with pyridostigmine. An immediate and dramatic improvement in the control of apnea, which was significantly reduced, was achieved.

The patient no longer required urgent hospitalizations due to apnea. According to parents’ observations, the number of episodes, which was two per day, decreased to five over 8 months. At the same time, it coincided with the little progress made in psychomotor development. The patient acquired the ability to crawl and sit down on his own for a short while. Parents also reported greater exercise tolerance during rehabilitation. At the same time, CPAP and oxygen therapy were used during the episodes, which could also have contributed to the observed improvement. The CPAP treatment was maintained during the patient’s sleep to prevent oxygen saturation drops at night. The NGS panel for neuromuscular disorders and familial dysautonomia (Invitae, SF, USA) showed no pathogenic variants in 110 known genes, including known genes related to congenital myasthenic syndromes. However, pyridostigmine treatment was maintained due to its positive effect on the number of apnea episodes.

The patient’s parents gave their informed consent for molecular testing and publication of clinical data, including photographs (without revealing the patient’s face). Table 1 summarises the clinical features of the patient at different ages and the results of laboratory, neuroimaging, and electrophysiological tests.

## 3. Discussion

The patient described in our paper is a 26 months old boy in whom a genetic test revealed the heterozygous variant c.1807G > C, p.(Glu603Gln) in the *NALCN* gene. This variant has not yet been reported in the ClinVar database or the literature to the best of our knowledge. As no variant carriers can be found in the gnomAD reference database, its allele frequency is unknown. This alteration affects the amino acid sequence, which is highly conserved among vertebrates. This amino acid position is also in close proximity to the functionally relevant S6 segment within the second pore-forming cation channel. Several pathogenic variants have been identified within or close to the S5 and S6 segments and occurred de novo in patients in the study by Chong et al. [3].

**Figure 1:**
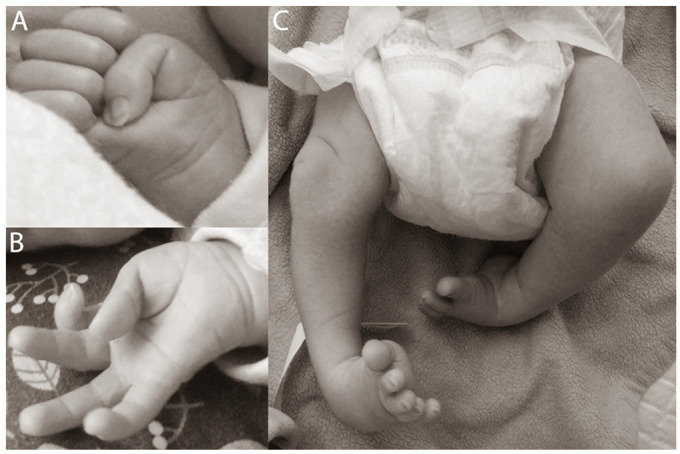
Clinical features in the proband during the neonatal period. The characteristic pattern of limb contractures presenting as (**A**,**B**) adducted thumbs, (**C**) feet, less obvious knees contractures, and equinovarus deformities of feet are shown.

Patients with CLIFAHDD syndrome have been described as presenting characteristic symptoms—congenital contractures of the limbs, dysmorphic features, hypotonia, neonatal respiratory distress, gastroesophageal reflux, and global developmental delay. We compared the clinical features of our patient with those described by Chong et al. (Table 2). Our patient, like the majority of other patients with CLIFAHDD, has a substantial delay in motor development (failure to achieve milestones, e.g., head position control), axial hypotonia, lack of speech development, respiratory insufficiency, recurrent apneas, gastroesophageal reflux, adducted thumbs, and severe deformities of feet. Adducted thumbs and common deformities of feet are essential features, but these may be overlooked, or their significance has been underestimated (Figure 1). At the present age of 2 years, the patient has begun to crawl, does not speak, has no proper non-verbal contact, and maintains normal eye contact. Therefore, we believe that the presence of a de novo heterozygous variant altering a conserved amino acid in close proximity to the S6 segment of the *NALCN* channel, together with characteristic neurologic phenotype, justifies the diagnosis of CLIFAHDD syndrome.

The most crucial differential diagnosis remains the exclusion of CMS, especially congenital myasthenic syndrome with episodic apnea (CMS-EA), caused by mutations in the *CHAT* gene, encoding choline acetyltransferase, or the *RAPSN* gene, encoding a postsynaptic protein, connecting and stabilizing acetylcholine receptors (AChR) at the neuromuscular junction [4]. Episodes of apnea and respiratory insufficiency are the hallmarks of *CHAT* pathogenic variants. Pathogenic variants in the *RAPSN* gene cause episodic respiratory insufficiency, arthrogryposis, and delayed motor development due to muscle weakness. In addition to genetic testing, edrophonium testing, repetitive nerve stimulation, or single-fibre electromyography can be utilized in preliminary diagnosis. This remains important due to the expansion of the genetic basis with several more recently described CMS genes associated with CMS-EA [5].

In 1989 Smith et al. described a group of superficial neurons in the medulla oblongata forming retrotrapezoid nucleus (RTN) [6]. RTN was localized under the facial motor nucleus, showing projections to the ventral respiratory column. Further studies established that the RTN integrated information that was transmitted to the brainstem central pattern generator (CPG) via tonically active glutamatergic neurons with Phox2b expression [7,8,9]. The CPG controls lungs ventilation and, therefore, the gas exchange process [7].

We currently know that the background activity of ion channels provides neuronal excitability [10]. Among these channels, *NALCN* protein forms a voltage-independent, nonselective cation channel generating so-called “leak”, which, under physiological conditions, is mainly based on the intracellular movement of sodium [11]. Mice lacking the expression of the *NALCN* showed hyperpolarization of the resting membrane potential of hippocampal neurons, which decreased their firing rate [12].

The functional outcome of *NALCN* knockdown in the retrotrapezoid nucleus was lower CO_2_-elicit neuronal activation, resulting in a decreased breathing process [12]. Furthermore, those *NALCN*-null mice died within 24 h of birth due to a disrupted respiratory rhythm, characterized by frequent and profound apneas. Thus, the *NALCN* channel present in the CO2/H-sensitive neurons of the mouse RTN is crucial for breathing regulation [13]. Importantly, *NALCN* is highly conserved in mammals, with a 96% sequence identity between humans and rats [14]. However, the *NALCN* is an orphan channel in humans, and most aspects of channel gating, ion selectivity, or pharmacology remain unknown.

Pyridostigmine is a carbamate acetylcholinesterase (AChE) inhibitor, which is widely used to treat myasthenia gravis [15]. This is due to the pyridostigmine peripheral mechanism of action that increases acetylcholine (ACh) transmission at synaptic junctions equipped with nicotinic or muscarinic receptors. In the physiological state, due to the positively charged ammonium group, pyridostigmine cannot freely cross the blood–brain barrier (BBB) [16].

However, some studies reported that, under conditions in which the BBB is disrupted (like acute stress), pyridostigmine penetrates the brain [17]. It is worth noting that several studies failed to replicate stress-induced acetylcholinesterase inhibition [18], but some of them do confirm the expected decrease in brain AChE P [19]. Pyridostigmine was further reported to modulate cortical excitability and induce alterations in gene expression [20].

One of the reports provided more evidence of this, showing that pyridostigmine affects CA1 pyramidal neurons [21]. Furthermore, it was found that pyridostigmine can mediate its effects via increased free ACh levels due to AChE inhibition. ACh activates muscarinic receptors, presumably in presynaptic localization, and raises glutamate release. Interestingly, it was also proven that stress is associated with long-lasting hypersensitivity to acetylcholine [22].

Other reports have suggested that pyridostigmine directly interacts with muscarinic receptors [23]. Interestingly, the *NALCN* encodes a current activated by M3 muscarinic receptors in a pancreatic b-cell line [24]. Another piece of research proposed that pyridostigmine’s central effects were due to indirect mechanisms emerging from a peripheral pathway [16]. Therefore, pyridostigmine has a central effect and may affect *NALCN* channels irrespective of the route of action.

Studies reported that mapping some of the known pathogenic variants to a model of *NALCN* indicated that they possibly affect the gating function of *NALCN* [25].

## 4. Future Directions

Hence, we believe that the clinically demonstrated efficacy of pyridostigmine in the presented patient has a justified molecular basis. In our considerations, we have indicated one example of the influence mechanism on retrotrapezoid nucleus neurons. By directly or indirectly influencing mutant *NALCN* channels, pyridostigmine is likely to restore their functionality to some extent.

## 5. Conclusions

Unfortunately, even an accurate diagnosis will not change the fact that, currently, there is no recommended and effective form of treatment for CLIFAHDD syndrome. Therefore, it seems all the more important to further analyze the unexpected efficacy of pyridostigmine in other patients with the same condition and try to explain the beneficial role of pyridostigmine in central apneas. This section is mandatory.

## Figures and Tables

**Table 1:** Clinical characteristics of the patient with CLIFAHDD syndrome.

Pregnancy	2nd Pregnancy, 2nd Delivery in 36 Hbd. Birth Weight 2920 g, Apgar 10. Polyhydramnios (Third Trimester), Diabetes Mellitus.
Perinatal period	Clubfoot (manual redresion, plaster casts were used). Adducted thumbs.
MRI of head	1st MRI (perinatal period)—normal. 2nd MRI (12 month)—slight enlargement of ventricular and subarachnoid space.
EMG	Failed to perform a full study. Routine examination showed no abnormalities.
EEG	1st EEG (perinatal period) and 2nd EEG (12 month)—in normal range.
Laboratory tests	GC/MS profiling of urinary organic acids, cerebrospinal fluid, test for congenital metabolic defects performed by tandem mass spectroscopy (perinatal)—normal.
Cardiological examination	ECG, ECHO—normal.
Pulmonary examination	Chest X-ray, bronchofiberoscopy—normal.
Gastroenterological examination	Gastroesophageal reflux
Orthopedic examination	Distal arthrogryposis
Dysmorphologic examination	Adducted thumbs, pits over large joints (knees, elbows), equinovarus feet, hypotonic face, without dysmorphic features.
Neurological examination	At birth: normal. At 2 weeks of age: No control of the head position in the traction test, no spontaneous control of the head position, no grasping of objects with hands. However, eye contact with the patient and cognitive development were normal. At 7.5 months of age: Psychomotor delay, no speech, the patient was lying down and he could turn sideways on his own, started to grasp objects, no control of the head position. Decreased muscle tone in the long axis with correct muscle tone in limbs. At 12 months of age: Alternating divergent strabismus present. Muscle tonus was still reduced within the trunk. Tendon reflexes were symmetrical, no neurological pathological signs were found. As before, there was no spontaneous control of the head position and during the traction test. In the horizontal position, spontaneous limb movements with the correct pattern were present. His thumbs were adducted. The patient was still lying down, and he could only turn sideways on his own. He maintained normal eye contact. After starting pyridostigmine treatment: Spontaneous improvement in head position control and in traction test. Patient began to crawl and sit alone.
Apnea episodes	At the age of 2 weeks: First episodes of skin graying, decreased muscle tension and rolling back of eyes. At the age of 3 months: The frequency of episodes increased. Initially, episodes began with a decreased muscle tone, which subsequently increased. Loss of consciousness was also present. Episodes lasted up to one minute. At the age of 12 months: Symptoms worsened. Episodes lasted over 3 min, with loss of consciousness and defecation. Choking has been observed several times. Episodes were provoked by crying or medical procedures. At night, O2 saturation drops to 70 percent without accompanying clinical symptoms. After starting pyridostigmine: Reduction in the number of apnea attacks. There were no episodes requiring urgent hospitalization or respiratory resuscitation. Due to the nocturnal O2 saturation reduction, overnight CPAP was recommended.
Genetic examination	Karyotype: 46, XY. Array-CGH—normal. Sanger sequencing of the entire coding sequence of the *ELP1* gene—normal. MLPA od the *SMN1* gene—normal. The single WES study (Cegat, Tübingen, Germany) showed one possibly pathogenic variant: c.1870G > C, (p.Glu603Gln) in the *NALCN* gene. Further Sanger sequencing in the patient‘s parents indicated that the variant aroused de novo.

**Table 2:** The comparison of the clinical symptoms described by Chong et al. [3] with the patient’s symptoms with a novel c.1807G > C, p.(Glu603Gln) variant in the *NALCN* gene.

Clinical Features	Patients Described by Chong et al. [3]	Patient Reported by Us
Downslanting palpebral fissures	10/14	−
Strabismus	7/14	+
Esotropia	3/714	+
Broad nasal bridge	14/14	−
Anterverted nasal tip	12/14	−
Large nares	14/14	−
Short columella	14/14	−
Long philtrum	12/14	−
Micrognathia	13/14	−
Pursed lips	9/14	−
H-shaped dimpled chin	8/14	−
Deep nasolabial folds	12/14	−
Full cheeks	13/14	−
Camptodactyly	14/14	+
Ulnar deviation	14/14	−
Adducted thumbs	14/14	+
Calcaneovalgus deformity	8/14	+
Club foot	8/14	+
Hip contractures	11/14	+
Elbow contractures	7/14	−
Knee contractures	9/14	−
Scoliosis	6/14	−
Short neck	10/14	+
Cognitive delay	11/14	+
Speech delay	12/14	+
Motor delay	14/14	+
Seizures	2/14	−
Hypotonia	7/14	+
Respiratory insufficiency	8/14	+
Abnormal respiratory pattern in newborn period	9/14	−
Excessive drooling	4/14	+
Gastroesophageal reflux disease (GERD)	9/14	+
Constipation	5/14	−
Hernia	9/14	−
